# New Remedies

**Published:** 1850-09

**Authors:** 


### NEW REMEDIES.

Dr. Debow, of Hartsville, Tennessee, mentions that a medical friend of his has been experimenting for some time past with the berries of the *Cornus Florida*, and that he has obtained from them an oil and an extract which he believes will be found to possess active remedial qualities. The gentleman is still engaged in the research, and promises to give us the results of it when he has made more extensive trials with the new remedies. We shall expect his communication with some interest, persuaded as we are that there are valuable medicinal properties in this plant which are not generally appreciated. Y.

